# *Toxoplasma gondii* merozoite gene expression analysis with comparison to the life cycle discloses a unique expression state during enteric development

**DOI:** 10.1186/1471-2164-15-350

**Published:** 2014-05-08

**Authors:** Michael S Behnke, Tiange P Zhang, Jitender P Dubey, L David Sibley

**Affiliations:** Department of Molecular Microbiology, Washington University School of Medicine, 660 S. Euclid Ave., St Louis, MO 63110 USA; United States Department of Agriculture, Agricultural Research Service, Beltsville Agricultural Research Center, Animal Parasitic Diseases Laboratory, Building 1001, Beltsville, MD 20705-2350 USA

**Keywords:** *Toxoplasma gondii*, Merozoite, Enteric stages, Definitive host, Life cycle, Parasite, Gene expression

## Abstract

**Background:**

Considerable work has been carried out to understand the biology of tachyzoites and bradyzoites of *Toxoplasma gondii* in large part due to *in vitro* culture methods for these stages*.* However, culturing methods for stages that normally develop in the gut of the definitive felid host, including the merozoite and sexual stages, have not been developed hindering the ability to study a large portion of the parasite’s life cycle. Here, we begin to unravel the molecular aspects of enteric stages by providing new data on merozoite stage gene expression.

**Results:**

To profile gene expression differences in enteric stages we harvested merozoites from the intestine of infected cats and hybridized mRNA to the Affymetrix Toxoplasma GeneChip. We analyzed the merozoite data in context of the life cycle by comparing it to previously published data for the oocyst, tachyzoite, and bradyzoite stages. Principal component analysis highlighted the unique profile of merozoites, placing them approximately half-way on a continuum between the tachyzoite/bradyzoite and oocyst samples. Prior studies have shown that antibodies to surface antigen one (SAG1) and many dense granule proteins do not label merozoites: our microarray data confirms that these genes were not expressed at this stage. Also, the expression for many rhoptry and microneme proteins was drastically reduced while the expression for many surface antigens was increased at the merozoite stage. Gene Ontology and KEGG analysis revealed that genes involved in transcription/translation and many metabolic pathways were upregulated at the merozoite stage, highlighting unique growth requirements of this stage. To functionally test these predictions, we demonstrated that an upstream promoter region of a merozoite specific gene was sufficient to control expression in merozoites *in vivo*.

**Conclusions:**

Merozoites are the first developmental stage in the coccidian cycle that takes place within the gut of the definitive host. The data presented here describe the global gene expression profile of the merozoite stage and the creation of transgenic parasite strains that show stage-specific expression of reporter genes in the cat intestine. These data and reagents will be useful in unlocking how the parasite senses and responds to the felid gut environment to initiate enteric development.

**Electronic supplementary material:**

The online version of this article (doi:10.1186/1471-2164-15-350) contains supplementary material, which is available to authorized users.

## Background

Intracellular parasites represent a significant portion of human disease burden throughout the world. The apicomplexan parasite *Toxoplasma gondii* is one of the most successful intracellular parasites and it is estimated up to a third of the human population has been infected [1]. This high infection rate results in approximately 1.5 million new infections in the U.S. per year. Fortunately, most infections do not result in debilitating symptoms as individuals with healthy immune systems are able to control the growth of the parasite, yet they are generally not able to eliminate chronic infection. Toxoplasmosis has been an indicator disease for patients suffering from complications of AIDS since the advent of the HIV viral epidemic [2]. Unborn babies can become infected in mothers who convert during pregnancy, resulting in 400–4,000 new congenital toxoplasma infections in the U.S. per year [3]. Also, the parasite can thrive in immune privileged areas of the eye, resulting in approximately 5,000 symptomatic ocular toxoplasmosis cases in the U.S. every year [4]. Major routes of human infection are via either the ingestion of undercooked infected meat or the accidental ingestion of oocysts shed into the environment, for example, by gardening or cleaning cat litter. Recent estimates by the CDC indicate that of known etiological agents, toxoplasmosis is the fourth leading cause of hospitalization and the second leading cause of death by foodborne illness in the U.S today [5]. The ability of Toxoplasma to infect such a large number of individuals, approximately 30 million in the U.S., results in meaningful disease burden in those individuals where the parasite circumvents normal modes of control [6].

**Figure 1:**
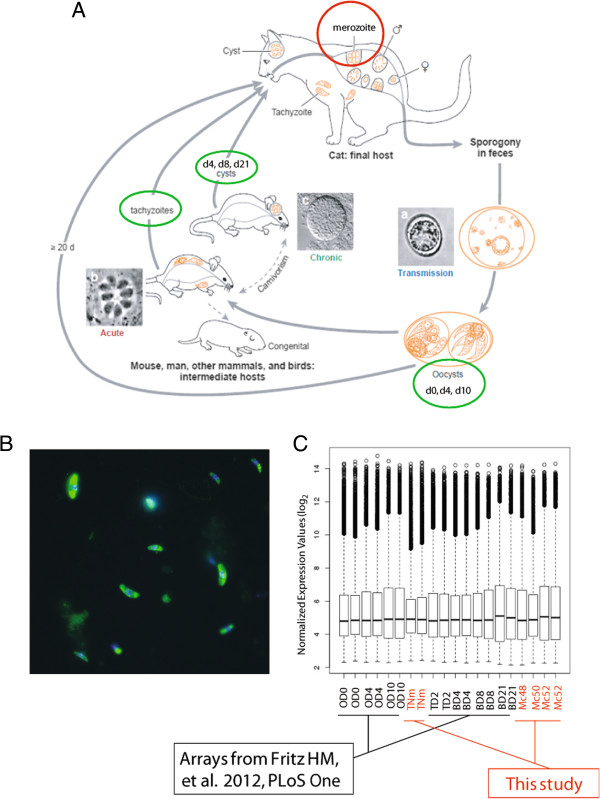
**Life cycle of** ***Toxoplasma gondii*** **, and the harvest and hybridization of merozoite mRNA. (A)** Life cycle of *Toxoplasma gondii* [58]. Green circle highlights stages for which microarray expression data exists [27]. Red circle highlights merozoite stage harvested for this study. **(B)** We obtained intestines from three cats (c48, c50, c52) that had been orally infected 5–6 days previously with the type II parasite TgNmBr1strain in order to isolate merozoite stage parasites. To determine that the purified forms were indeed Toxoplasma, a portion of sample c52 was labeled with mouse α-Me49 (Alexa Fluor 488, yellow). DAPI stained nuclei (blue). **(C)** mRNAs for two merozoite c52 (Mc52) samples and one for each of the c48 (Mc48) and c50 (Mc50) samples were labeled using the Ambion MessageAmp kit and hybridized to Affymetrix Toxoplasma GeneChips. In order to control for possible strain specific expression we also hybridized two TgNmBr1 tachyzoite mRNA samples (TNm). Data were analyzed in combination with the recently published dataset covering oocyst (Day 0 (OD0), 4 (OD4), 10 (OD10)) to tachyzoite (TD2) to bradyzoite (Day 4 (BD4), 8 (BD8), 21 (BD21)) development in the type II M4 strain [27]. Boxplots of the RMA normalized data show similar distributions and median values across the samples.

There are two aspects of the Toxoplasma life cycle that allow it to be so prevalent, the ability to infect a vast number of intermediate hosts and the ability to produce millions of environmentally resistant oocysts through a single infection of a cat, the definitive host [7]. *Toxoplasma gondii* has been found to infect virtually every warm blooded animal that has been assessed [8]. Because of this, many animals used for food consumption have dormant bradyzoite cysts in their tissues, and if not properly cooked, these parasites can be passed to the consumer leading to infection. This points out a unique feature of the *T. gondii* life cycle, as permissive infection of intermediate hosts following ingestion of tissue cysts [9] is normally only infective for the definitive host in related apicomplexan parasites [10]. Upon infection, slow growing bradyzoites differentiate into rapidly growing tachyzoites that continue to divide and rupture infected cells causing cellular damage. Once the immune system is triggered, parasite growth is controlled, and the parasite differentiates back into the dormant bradyzoite cyst effectively hiding from the immune system, often in immune privileged areas such as the brain. This bradyzoite-tachyzoite-bradyzoite progression can result in repeated rounds of infection in multiple intermediate hosts, without the need for sexual transmission. In order to complete the life cycle and differentiate into sexual stages, the parasite must pass through the gut of a felid. Although, fortunately, cat predation of humans is extremely rare, cats can be exposed to the parasite by ingesting other infected intermediate hosts, such as mice. Once bradyzoites pass into the intestine of a cat they can differentiate through the coccidian stages of development, or enteric stages, progressing from asexual merozoite forms into sexual stage micro- and macrogametocytes that eventually fuse to form oocysts which are shed into the environment where they mature into sporulated oocysts. Sporulated oocysts can then be ingested by intermediate hosts, where infectious sporozoites liberated from oocysts differentiate into tachyzoites completing the life cycle (Figure 1A) [7, 11].

Much work has been carried out to understand the biology of the asexual stages, tachyzoites and bradyzoites, which occur in the intermediate host. One reason for this is the relative ease in culturing these stages in the laboratory, both in tissue culture and the mouse model. Tachyzoites grow well in various cell lines, such as human foreskin fibroblasts (HFFs), and bradyzoites can be induced *in vitro* [12, 13] via cellular stresses [14, 15] or *in vivo*. These culturing methods have allowed researchers to develop an extensive molecular toolbox for Toxoplasma, resulting in an increased understanding of its biology including host cell invasion and egress [16], how the parasite modulates the intracellular environment [17], and the identification and characterization of virulence factors that allow the parasite to evade host innate immune defenses [18]. Culturing methods for stages beyond the bradyzoite, the merozoite and sexual stages, have not been developed, hindering the ability to study a large portion of the parasites life cycle and restricting such work to a few laboratories with the resources to house cats.

There are several reports describing various aspects of the enteric stages of *T. gondii* within felid intestinal cells [19–22]. These studies used electron microscopy to observe ultrastructural features or performed immunohistochemistry (IHC) to determine the labeling of parasites with various antibodies, useful in characterizing the expression of a limited number of proteins in enteric stages. Another report demonstrated the feasibility of isolating merozoite stage parasites from the intestinal mucosa of infected cat intestine [23] and showed these parasites illicit unique antigenic responses [24]. Based on this report, we undertook isolation of merozoites for genome wide expression profiling study in order to identify merozoite-specific genes.

## Results

### Isolation of merozoite stage parasites and mRNA hybridization

To provide insight into gene expression of the enteric stage, we harvested intestines of three cats (cat numbers: c48, c50, and c52) infected with the type II parasite, TgNmBr1 [25]. The harvesting process resulted in the removal of the majority of host cells and debris, leaving crescent shaped merozoite parasites. To determine that the purified parasites were indeed *T. gondii*, a portion of the sample was fixed and stained with sera from mice immunized with type II Me49 parasites (Figure 1B). Indeed, the crescent shaped purified parasites were labeled with the *Toxoplasma* specific antibody. Purified parasites from all three felid intestines were processed for mRNA isolation and hybridized to the Affymetrix Toxoplasma GeneChip [26] (array sample names: Mc48, Mc50, and Mc52). In order to place the newly acquired merozoite gene expression in context of the life cycle, the data were analyzed in combination with a recently published dataset by Fritz HM et al. 2012 [27] covering day 0, 4, 10 oocyst (array sample names: OD0, OD4, OD10), day 2 tachyzoite (TD2), and day 4, 8, 21 bradyzoite (BD4, BD8, BD21) development (Figure 1C). Although the parasites used in the present study (TgNmBr1 strain) and the Fritz HM *et al.* study [27] (M4 strain) are both clonal type II strains, they are of different origins; the former is from a guinea fowl from Brazil, and the latter is from a sheep in United Kingdom. To control for any strain specific differences in identifying merozoite specific genes, we also hybridized mRNA from TgNmBr1 tachyzoites (TNm). Plotting of RMA normalized values for all the samples used in the analysis demonstrated that the medians of the distributions converge and that the ranges of extreme values across all samples were similar, lending weight that the samples from the current study and the Fritz HM *et al.* [27] study are comparable (Figure 1C).

### Life cycle gene expression analysis

**Figure 2:**
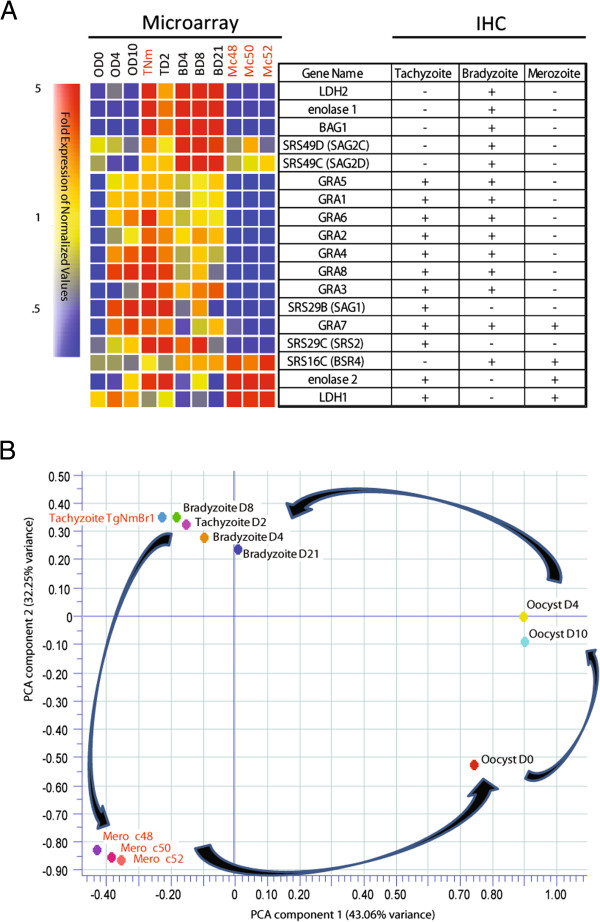
**Merozoite gene expression supports published data and forms a distinct grouping when compared with other life cycle stages. (A)** Heat map of expression (left, this study) and binary indication of labeling by IHC (right) for genes that were assessed for merozoite stage expression previously [20]. There was excellent agreement when the two studies are compared, where, for example, gene products that did not label by IHC at the merozoite stage (-) were not expressed as indicated by the microarray data (blue). **(B)** Principal component analysis (PCA) highlighted the unique profile of the merozoite samples placing them approximately half-way on a continuum between the tachyzoite/bradyzoite and oocyst samples. The first two components described a combined 75% of the variance across the samples.

In order to verify that the purified parasites were enteric stage merozoites, and not morphologically similar tachyzoites, we used as a control a gene list for which the expression status at the merozoite stage had been determined using immunohistochemistry (IHC) [20]. Comparison of the IHC data on protein expression showed excellent agreement with the merozoite gene expression data (Figure 2A). For example, Ferguson DJ [20] reported that antibodies to surface antigen 1 (SAG1) and many dense granule proteins (GRAs) did not label merozoites, and the microarray data from all three merozoite samples (Mc48, Mc50, and Mc52) confirmed that these genes were not expressed at this stage. Likewise, the expression pattern was consistent in both experiments for genes that were expressed in merozoites, such as *enolase 2* and *LDH1*, which were not expressed in bradyzoites but were expressed in the tachyzoite and merozoite stages (Figure 2A). The agreement between these datasets support the conclusion that the parasites harvested from the infected felid intestines are indeed merozoites.

To determine the major trends across the samples we conducted principal component analysis (PCA) that highlighted the uniqueness of the merozoite samples (Figure 2B), clustering them approximately half-way on a continuum between the tachyzoite/bradyzoite and oocyst samples. The distinct grouping of the merozoite samples was not the result of strain specific differences as the tachyzoite TgNmBr1 strain sample (TNm), the same strain used to generate the merozoite parasites, grouped with the tachyzoite M4 strain Day 2 sample (TD2) Interestingly, the PCA analysis revealed that compared to the merozoite and oocyst stages, the tachyzoite and bradyzoite stages were quite similar and they all group together. Although studies have shown there are gene expression differences between these two stages [28–30], the PCA of global expression profiles indicates that bradyzoites resemble dormant tachyzoites when taken in context of the whole life cycle. Lastly, the circular progression of samples, from the OD0 to OD4/OD10 cluster, to the tachyzoite/bradyzoite cluster, and finally to the merozoite cluster, follows the progression of the life cycle (Figures 1A and 2B).

We identified life cycle regulated genes by performing an analysis of variance test (ANOVA p-value cutoff .05) on genes with expression above background levels in at least one sample. This analysis resulted in the identification of 5,969 genes, a large proportion (73.4%) of the genes in the *Toxoplasma* genome. There were 1,571 non-expressed genes (19.3%) for which expression was at or below background in all samples, and 591 non-regulated genes (7.3%) that were not significantly differentially expressed between the samples. Although the life cycle regulated gene set (5,969) captures most of the *T.gondii* genome, a fifth of the genes represented on the array did not have detectable expression. Some of these non-expressed genes may represent stage specific genes for stages which we do not have expression data, such as the micro- and macrogametes.

**Figure 3:**
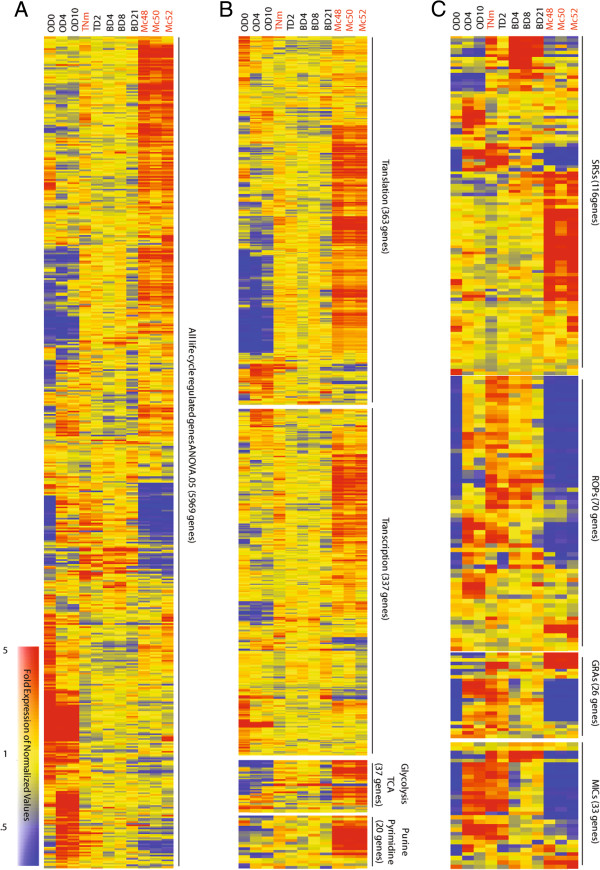
**Clustering of significantly regulated life cycle gene groups. (A)** Heatmap of life cycle regulated genes. A total of 5,969 genes were found to have significant expression differences (ANOVA .05). **(B)** Heatmap of cell growth related genes. Many genes with growth and/or metabolism related Gene Ontology were upregulated at the merozoite stage. **(C)** Heatmap of Apicomplexa specific genes. The ROP, GRA and MIC gene families were downregulated as a class and many SRS genes were upregualted upon entering the enteric stages.

The regulated genes (5,969) were clustered into a heatmap revealing that the merozoite samples have a unique gene expression profile with hundreds of genes increased or decreased as compared to the other life cycle stages (Figure 3A). This pattern was consistent for all three merozoite samples. Many different expression patterns emerge from the heatmap (Figure 3A), but one major theme is the large number of genes that were differentially regulated at the merozoite and oocyst stages as compared to the tachyzoite and bradyzoite stages. When a 2.5 fold expression cutoff was applied on the Mc52 sample, 1,918 of the regulated genes (32.1%) were specifically regulated at the merozoite stage, with 1,249 being upregulated and 669 genes downregulated at this stage. To identify biological processes that may be life cycle regulated, we performed standard Fisher’s exact tests on overlapping genes between the regulated gene list (5969) and a comprehensive set of lists based on Gene Ontology (GO). The two most significant GO lists were cell growth & maintenance (p-value 1.92e-251) and metabolism (p-value 5.7e-153). More specifically, within these inclusive GO categories, we found three sub-categories of interest; Translation (p-value 2.39e-52), Transcription (p-value 2.9e-20), and Glycolysis & TCA (p-value 5.75e-5) (Figure 3B). The heat maps show that many genes within these GO categories are uniquely upregulated at the merozoite stage (Figure 3B). Especially striking were the large number of genes upregulated in the Glycolysis/TCA and Purine/Pyrimidine metabolic pathways. To further extend the metabolic analysis we mapped the expression of all the regulated genes for which there are KEGG annotations (KEGG genome T01093) to the KEGG metabolic pathways map (tgo01100), creating maps for each life cycle stage (see Additional file 1). Two major themes were revealed in these metabolic maps. First, many pathways started from a downregulated state (blue) at the OD0 sample, progressively increased in expression through OD4 and OD10, exhibited steady state expression in the tachyzoite and bradyzoite samples (yellow), and, finally, pathways throughout the metabolic map were upregulated at the merozoite stage (red). Secondly, the expression of the fatty acid biosynthesis (FAB) and degradation (FAD) pathways oscillates throughout development (two repeating sets of vertical pathways at left of center in Additional file 1: FAB left set, FAD right set). FAB was downregulated in the OD0, OD4 and OD10 samples, steady state in tachyzoite and bradyzoite, and upregulated in the merozoite. In an opposite manner to FAB, FAD was largely downregulated in the invasive stages, the tachyzoite and merozoite, and upregulated in the encysted stages, the oocysts and bradyzoite. The regulation of the FAD pathway through life cycle development may provide the encysted stages a poised state to utilize fatty acid stores that are present, for example, in the oocyst [31]. Overall, the GO and KEGG analysis demonstrated that the merozoite has an elevated gene expression state for cell growth and metabolic related genes, indicating unique growth requirements of this stage.

We also looked at the expression of parasite-specific gene families in the merozoite stage. As seen in Figure 2A, many of the annotated GRA genes (50%) are not expressed at the merozoite stage (Figure 3C). Genes in two other parasite specific gene families, the rhoptry (ROP) (61%) and microneme (MIC) (60%), similarly lacked expression in the merozoite stage. For example, none of the annotated rhoptry neck (RON) or apical membrane (AMA) genes, important for parasite invasion in intermediate stages, were expressed at the merozoite stage. One RON paralog, *RON4L1* [27], had slight expression in the merozoite, but the expression was downregualted as compared to the tachyzoite/bradyzoite stages. Likewise, genes that have been shown to be important for host immune evasion, such as ROP18, ROP5, ROP16, GRA15, and GRA24 [18, 32], were not expressed. Although essentially half of the genes in these gene families were not expressed as merozoites, others had low constitutive expression, and a few were upregulated. Those that exhibited upregulated expression include: GRA family (*DG32 antigen*, *DG32 protein*, *GRA12* and *NTPase I*), ROP family (*ROP21*, *ROP32*, *ROP33*, *ROP36*, *TGME49_281790* (kinase), and *TGME49_249470* (rhoptry kinase)) and MIC family (*TGMe49_200270* (PAN/Apple domain), *TGME49_275800* (SRP72), *TGME49_286150* (PAN/Apple domain), and *TGME49_254430* (microneme)), but even most of these were low abundance expressed genes. See Additional file 2 for specific genes and expression values. The downregulated expression state upon entering the enteric stages for many members of these gene families is unexpected as many have been shown to play a role in host-parasite interactions in intermediate hosts, for example invasion [33] or host immune modulation/resistance [18], and suggests they are not needed during enteric development. Converse to the GRA, ROP, and MIC families, many members of the parasite surface antigen gene family (SRS) were upregulated (44.8%) upon differentiating into the first of the enteric stages as a merozoite (Figure 3C). Although developmentally regulated expression of various SRS genes has been shown for other stages, in comparison, quite a large number were upregulated at the merozoite stage. Members of the SRS gene family are involved in parasite adhesion, invasion, and virulence, possible roles they may have in allowing the parasite to develop effectively in the cat intestine [34].

### Timing and control of life cycle gene expression

**Figure 4:**
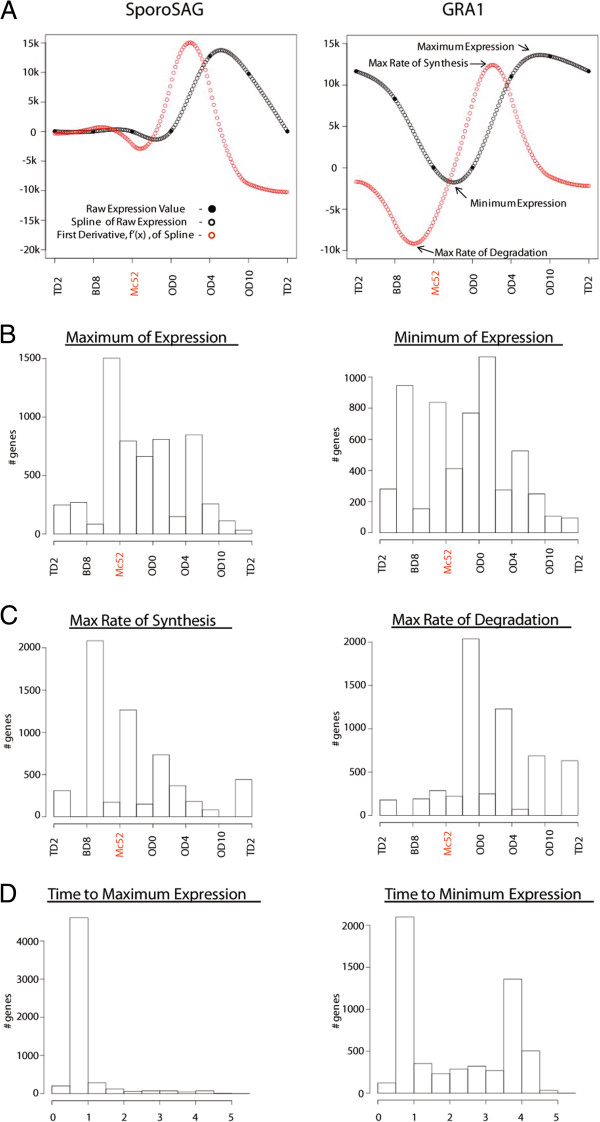
**Timing of life cycle gene expression. (A)** Example spline and *f’(x)* curves for two genes, SporoSAG and GRA1. The maximum and minimum expression values were determined from the spline curve (arrows black curve). The maximum rate of synthesis and degradation were determined from the *f’(x)* of the spline curve (arrows red curve). **(B)** Number of genes with maximum or minimum expression across the life cycle calculated from spline curves for all 5,969 life cycle regulated genes. **(C)** Maximum synthesis and degradation rates across the life cycle calculated from *f’(x)* of spline curves for all 5,969 life cycle regulated genes. **(D)** For each of the 5,969 regulated genes the expression value **(B)** was subtracted from the respective synthesis value **(C)** to determine time to maximum or minimum expression. Histograms displayed at half stage intervals for B-D.

Determining peak and valley points of expression for gene profiles that span continuous data sets has been useful in gaining insight into parasite biology [35, 36]. By generating spline curves from expression values for all regulated genes (5,969) we were able to determine the points of maximum and minimum expression for each gene. As an example, we have graphed the curves for the *SporoSAG* and *GRA1* genes (Figure 4A). The points of maximum expression for *GRA1* and *SporoSAG* were both between the sporulated oocyst OD4 to OD10 samples, and *GRA1* had a deep valley of minimum expression between the merozoite and unsporulated oocyst, which correspond to the known roles for these genes [20, 37]. We ordered the maximum and minimum values for all regulated genes according to the life cycle and displayed the distribution as a histogram (Figure 4B). There was a large shift in the number of genes at peak expression at the merozoite stage, Mc52, corresponding to the stage where the largest number of genes reached maximum expression. There were also a large number of genes that peaked throughout the oocyst stages, OD0 and OD4, while fewer genes peaked at OD10, due to its similarity with OD4 (Figure 3A). By far, the tachyzoite and bradyzoite stages had the fewest number of genes peaking at those stages. A slightly different distribution emerged when genes were ordered by minimum of expression (Figure 4B). Many genes had minimal expression throughout the enteric stages, but there were also a large number of genes with minimum of expression between the tachyzoite and bradyzoite stages.

By calculating the maximum and minimum values of the first derivate (*f’(x)*) of the spline curve, one can determine the inflection points in the graph which correspond to the points of maximum rate of synthesis and degradation. For example, *GRA1* (Figure 4A) had an upwardly trending inflection point just after the OD0 sample corresponding to the maximum rate of synthesis just before the peak of expression between OD4 and OD10, and a downward trending inflection point between the BD8 and Mc52 samples indicating the maximum rate of degradation just before the gene is no longer expressed in the merozoite. There was a wave of synthesis throughout the life cycle starting with entry into the merozoite stage, Mc52, steadily tapering through the oocyst stages (Figure 4C). This progression largely mirrored the maximum expression distribution where maximum rates of synthesis (Figure 4C) precede maximum points of expression (Figure 4B) by half a stage. The distribution seen for maximum rate of synthesis was shifted one stage down the life cycle when genes were ordered by maximum rate of degradation (Figure 4C), reflecting the peak of expression that occurred between synthesis and degradation. Lastly, we determined the time, or number of stages, to maximum or minimum expression by subtracting the maximum or minimum expression value (Figure 4B) for each regulated gene from the corresponding rate value (Figure 4C). For the upregulation of gene expression, most genes reached peak expression very rapidly, within one stage (Figure 4D), indicating that once the cell commits to the rapid synthesis of a gene the expression peaks quickly in relation to the life cycle. This was also a result of the stage-specific expression for many genes. When genes were downregulated, many genes reached their minimum of expression rapidly, but there was also a group of genes that had a delayed minimum after the greatest rate of degradation, approximately four stages later (Figure 4D). This delayed grouping indicates that the minimum of expression for a large number of genes was just before the upregulation of that gene, as there are only 6 stages represented in this analysis.

**Figure 5:**
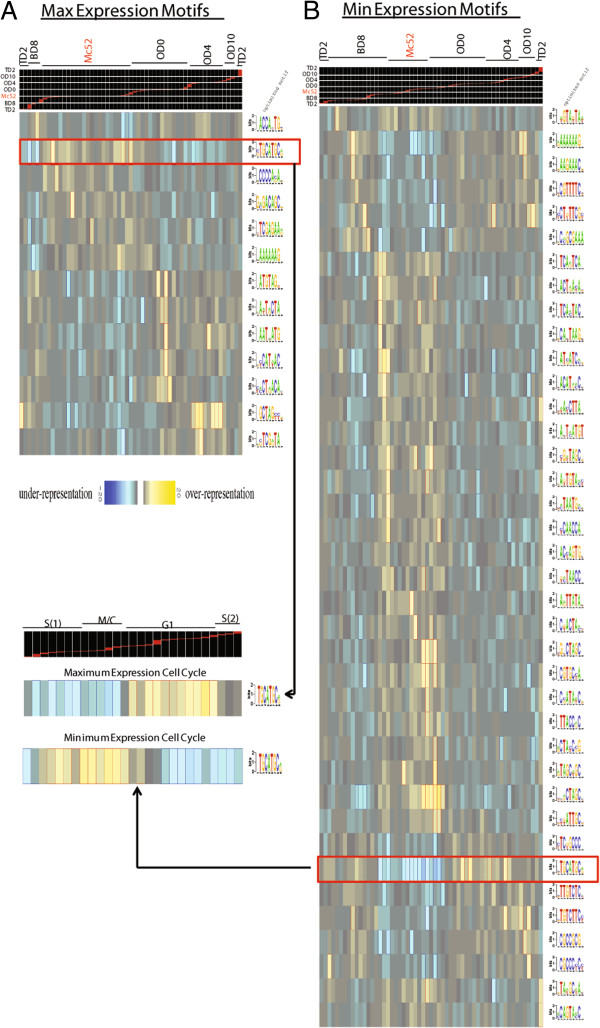
**Motif discovery in promoter regions of life cycle regulated genes. (A)** Life cycle regulated genes (5,969) were ordered by point of maximum expression and the FIRE software package was used to find motifs over- and under-represented in sequence 2,000 bp 5’ of the CDS start. Motifs over-represented at a particular point in the life cycle (yellow), those under-represented (blue). **(B)** Life cycle regulated genes were ordered by point of minimum expression and run in FIRE as in **(A)**. The TGCATGC motif found in both **(A)** and **(B)** was also a major motif found in the Toxoplasma cell cycle expression data set [35] where, for example, it was over-represented at G1 for genes with maximum expression at that phase (inset). No significant motifs were found across 100 FIRE runs when data in **(A)** or **(B)** were randomly ordered.

It has been demonstrated that promoter elements upstream of coding genes are critical for control of gene expression in *Toxoplasma*, for both constitutive and developmentally regulated genes, and that minimal *cis* elements provide regulatory specificity [28, 38–40]. Many of the life cycle regulated genes had dramatic periodicity of expression (Figure 3A and Figure 4), suggesting that co-regulated genes may share common regulatory elements. We ordered genes by maximum or minimum expression (Figure 4B) and examined the sequence 2,000 bp upstream of the CDS start to search for common regulatory elements using FIRE [41] (Figure 5). When genes were ordered by maximum expression (Figure 5A), 13 conserved motifs were found in promoter regions of regulated genes (no motifs were found across 100 permutations with the maximum expression data randomly ordered). The TGCATG motif that was over represented in genes that span the merozoite stage is a TRP2 *cis* element [42] and was also found to be a major motif in a previous cell cycle expression study [35], where TGCATG was overrepresented at the G1 phase of the cell cycle when genes were ordered by maximum expression (Figure 5A arrow to inset). Also, the GCTAGC motif overrepresented at the OD4 stage was found in the cell cycle study, and a recent report described an AP2 transcription factor, TgAP2-XI-5, that binds the corresponding GCTAGC motif [43]. TgAP2-XI-5 regulates hundreds of genes, including *ROP18*, which like many of the rhoptries, increased expression in the life cycle at OD4 from a non-expressed state at OD0 (Figure 3C). Lastly, the ACCA(A/C/T)TG motif, similar to the BAG1 *cis* element CCAGTA [28], was overrepresented at the BD8 stage, a cluster which contains the *BAG1* gene. Ordering genes by the minimum of expression (Figure 5B) found many motifs, 38, overrepresented at points that together span the whole life cycle (no motifs were found across 100 permutations with the minimum expression data randomly ordered). The TGCATGC and GCTAGC motifs were found in the minimum expression ordered data, along with many other motifs that represent possible *cis* elements. This pattern may reflect a role for repressor mechanisms constituting a major portion of gene expression control in the *Toxoplasma* life cycle. The FIRE analysis for the life cycle regulated genes (Figure 5) identified overrepresented motifs of known function (i.e., TGCATG, GCTAGC, and ACCA(A/C/T)TG) indicating that newly identified motifs may be functional *cis* elements. Additionally, motifs were only identified when data were ordered in a biologically relevant manner.

### Promoter control of merozoite specific expression

**Figure 6:**
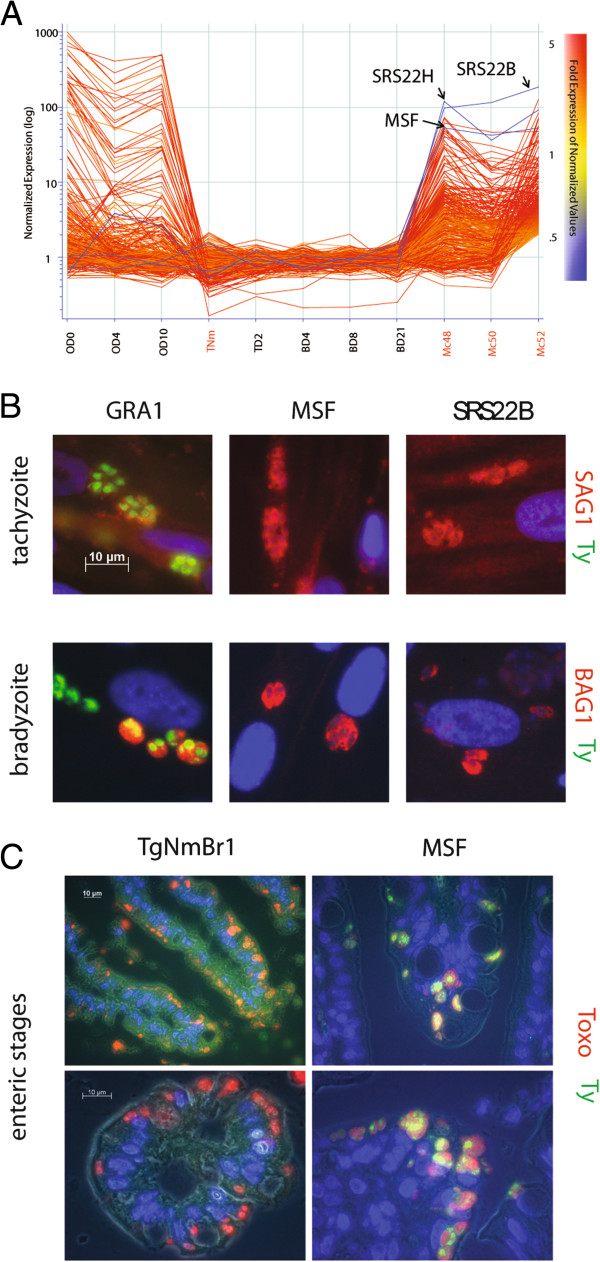
**Promoter control of merozoite specific gene expression. (A)** Genes upregulated specifically in the merozoite stage (color of individual gene expression profiles based on the Mc52 expression). Three candidate genes for use in cloning merozoite specific promoters are highlighted (blue). **(B)** Transgenic GRA1, MSF and SRS22B strains were assessed for expression of *DHFR-Ty* at the tachyzoite (α-Ty + Alexa Fluor 488, yellow and α-SAG1-Alexa Fluor 594, red) and bradyzoite (α-Ty + Alexa Fluor 488, yellow and α-BAG5 + Alexa Fluor 594, red) stages by IFA. Strong nuclear expression of *DHFR-Ty* was observed for the GRA1 strain and lacked expression for MSF and SRS22B strains at both stages, which tracks with the microarray expression data (Figure 4A and Figure 6A). **(C)** The MSF strain was assessed for enteric stage expression of *DHFR-Ty* in the felid intestine (α-Ty + FITC, yellow and α-RH-parasite + TRITC, red). Non-transgenic TgNmBr1 parasite was used as a negative control. Expression of *DHFR-Ty* was observed for the MSF strain in the enteric stage, confirming stage specific control of expression by the promoter.

**Figure 7:**
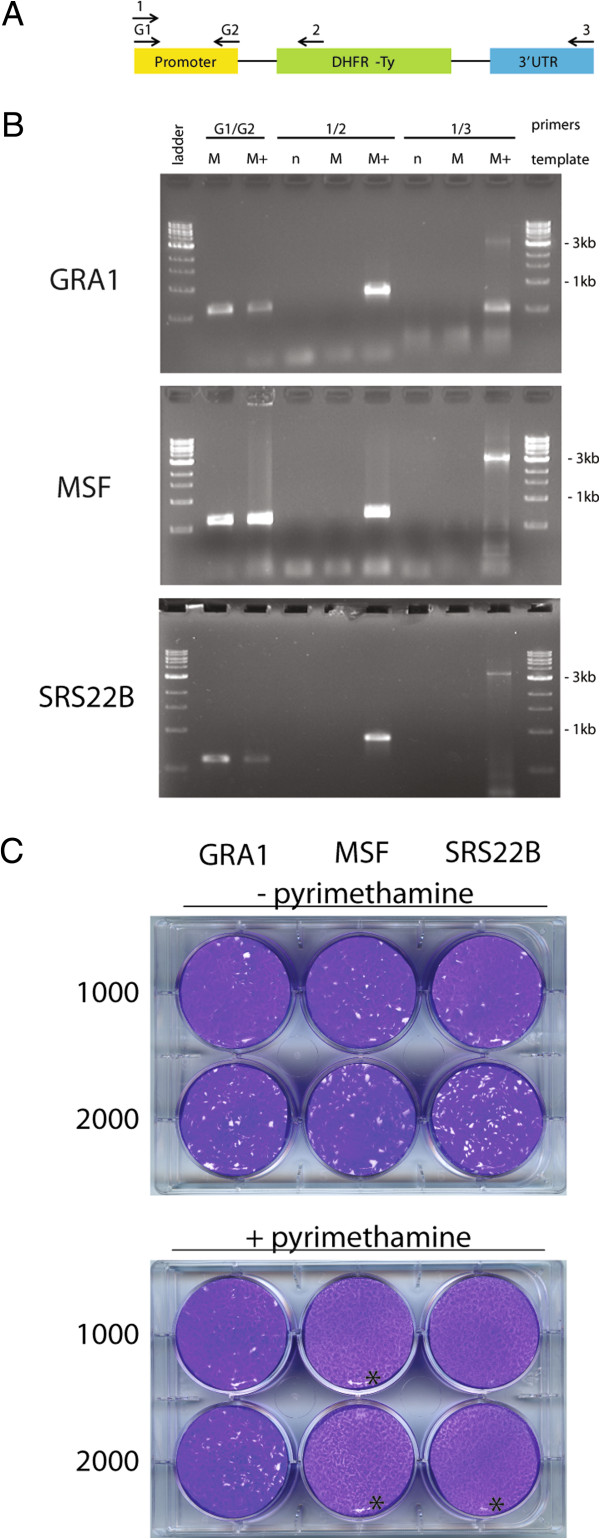
**Characterization of transgenic strains. (A)** Schematic of the DHFT-Ty expression cassette in the three pDEST-(*)p-DHFR-Ty plasmids and the locations of primers used in (B). **(B)** PCR analysis of transgenic Me49-Fudr clones to detect plasmid integration: G1 and G2 – GRA1p specific primers, ladder - 1 kb ladder (New England Biolabs), n (no template control), M (Me49 template), M + (Me49 transgenic clone template). **(C)** Growth of transgenic Me49-Fudr clones with or without the drug pyrimethamine. All three transgenic clones grew, creating plaques, in normal D10 media without drug (top). Only the Me49-GRA1p-DHFR-Ty clone grew in D10 + pyrimethamine (bottom, *indicates disturbance of the monolayer due to mechanical means, not parasite growth).

Given that promoters have been shown to control developmental gene expression in the intermediate tachyzoite and bradyzoite stages and that a large number of motifs were found associated with the life cycle expression data (Figure 5), we were interested to test if promoters were sufficient to control gene expression at the merozoite stage. As part of the experimental design we also sought to drive the expression of a drug resistance gene only at the merozoite stage. A list of 362 merozoite-specific genes was identified by restricting the life cycle regulated genes (5,969) to those that were not expressed in tachyzoite or bradyzoite stages but were upregulated in the merozoite (Figure 6A). The expression of many of these genes at the merozoite stage was quite high, some being regulated > 100 fold as compared to bradyzoites. For example, two of the SRSs, *SRS22B* and *SRS22H*, were among the highest expressed genes in this set, and *TGME49_201180*, annotated as a megakaryocyte stimulating factor (*MSF*), was the highest expressed annotated gene across all merozoite samples excluding the *SRS* genes. These three genes were chosen as candidates to use in cloning promoter regions into a Gateway plasmid construct containing the drug resistance *DHFR* gene tagged with a Ty epitope (*DHFR-Ty*). We also cloned the *GRA1* promoter (*pGRA1*) in front of the *DHFR-Ty* for use as a control, as *GRA1* is expressed in tachyzoites and bradyzoites and not in merozoites. All four constructs were individually electroporated in the Me49-Fudr parasites, as this is a type II parasite and has been used successfully in various genetic crosses, and after selection and cloning we were able to obtain transgenic parasites containing the p*GRA1*, p*MSF* and p*SRS22B* promoter constructs. The clones were screened by PCR for integration of the plasmid using primers that span the promoter of interest and the *DHFR* portion of the construct (Figure 7A & B). The effectiveness of gene expression control by these promoters and of the drug resistance *DHFR* gene was tested by plaque assay (Figure 7C). All three promoter strains grew equally as tachyzoites, creating plaques after 9 days of growth in HFF monolayers cultured with regular media lacking pyrimethamine (Figure 7C top). Only the *pGRA1* expressing strain grew to create plaques after 9 days in media containing the drug (Figure 7C bottom). The presence of plaques for the *pGRA1* expressing strain grown with pyrimethamine confirms that the *DHFR-Ty* gene confers drug resistance, and the absence of growth for either *pMSF* or *pSRS22B* expressing strains shows they lack *DHFR-Ty* expression and remain drug sensitive. Parasites expressing the *pMSF* and *pSRS22B* reporters were grown for up to 18 days in pyrimethamine and no growth was observed by plaque assay or when the monolayers were screened under the microscope (data not shown). We also conducted immunofluorescence assays (IFA) on the three strains to test for the expression of *DHFR-Ty* at both the tachyzoite and bradyzoite stages. As expected, only the *pGRA1* expressing strain had detectable Ty labeling at both stages, staining the nucleus of the cells (Figure 6B). Lastly, in order to test the developmental regulation of expression during enteric development, mature bradyzoite cysts of parasites harboring *pMSF* were generated in chronically infected mice and fed to a cat for the production of oocysts. At the first indication of shed oocysts, the intestine was harvested and processed for IHC. As a control we used an IHC prepared section of the intestine infected with the original TgNmBr1 strain that was used to harvest merozoite mRNA (Figure 1B). In contrast to tissue culture, where the *pMSF* driving expression of *DHFR-Ty* was not detected, parasites in the gut labeled brightly with α-Ty antibody (Figure 6C). No Ty labeling was seen in the TgNmBr1 sample (Figure 6C). The labeling pattern seen for the MSF strain in Figure 6 B & C matches what was predicted from the expression data (Figure 6A) and shows that upstream regions are sufficient to control expression of a merozoite gene. Much like developmental regulation in the intermediate stages, *cis* elements and specific factors that bind them to control gene expression are active in the enteric stages.

## Discussion

Here we describe the global gene expression of the merozoite stage of *Toxoplasma gondii* and analyzed this stage in context of the life cycle in combination with previously published array data from other life cycle stages [27]. We confirm that the expression patterns observed for the merozoite samples matched data on protein expression for enteric stage parasites [20]. We also substantially extend these prior studies by showing that many parasite specific gene families such as GRAs, ROPs and MICs were downregulated at the merozoite stage. Functions for many members of these families have been described for the intermediate stages of the parasite, and the regulation at the merozoite stage suggests that these genes are not needed during intraepithelial development in the definitive host. Interesting, genes known to be critical for moving junction formation and thus parasite invasion during the intermediate stages are not expressed in the merozoite. Although not much is known about how merozoites invade, the shared morphological characteristics of tachyzoites and merozoites suggests an active invasion process, which based on the expression data is not reliant on known RON/AMA interactions. Speciation of apicomplexan parasites may have occurred via evolutionary divergence of definitive hosts [44, 45]. Many members of the parasite-specific gene families are the result of gene duplication and expansion events, and those few members that are upregulated at the merozoite stage, the first stage the parasite differentiates into within the definitive host, may represent the ancestral copies from which the expanded intermediate stage expressed genes arose.

The merozoite occupies a unique place in the overall gene expression continuum of the *T. gondii* life cycle, clustering between tachyzoite/bradyzoite and oocyst samples as shown by PCA. It will be of interest to isolate other enteric forms of *Toxoplasma*, such as the micro- and macrogamete for global gene expression studies to determine if the missing forms will follow this progression, thus being placed between the merozoite and oocysts clusters. Additionally, a large number of genes were regulated when the parasites enter coccidian development. Many of these genes related to cell growth/maintenance (Translation and Transcription GO categories) and metabolic processes (Glycolysis and TCA Cycle GO categories) were upregulated at the merozoite stage. Different than the intermediate stages, the merozoite divides by processes similar to schizogony termed endopolygeny, where multiple daughters are generated before the plasma membrane resolves the individual parasites [46]. It is possible that the merozoite specific upregulation of cell growth and metabolism related genes is a consequence or even a requirement for the endopolygeny form of division. On the other hand, the elevated expression of growth and metabolism related genes is reminiscent of the Warburg effect, where, for example, cancer cells within hypoxic tumor microenvironments increase expression of metabolism related genes, such as glycolysis [47]. The merozoite preferably grows in the microaerobic environment of the small intestine and it may be the hypoxic nature of the small intestine [48] that partially explains the unique expression pattern of growth and metabolic related genes in the merozoite.

We also demonstrate that promoter control of gene expression is conserved at the merozoite stage. Quite a number of conserved motifs were found in co-regulated genes, whether when ordering genes by the maximum or minimum of expression. Identifying conserved over-represented motifs by ordering genes in this manner may indicate regulatory control for a particular motif. For example, the large number of motifs overrepresented throughout the life cycle when genes were ordered by the minimum of expression indicates that these motifs correspond to points in the life cycle when expression is at the lowest and thus may be associated with repressor mechanisms. Indeed, recently the AP2 transcription factor, AP2IX-9, was shown to operate as a repressor by inhibiting bradyzoite-specific gene expression [49], a role many of the 67 annotated AP2 genes, or other *cis* element binding proteins, in *Toxoplasma* may share. In addition to identifying conserved motifs associated with gene expression patterns, we confirm that a merozoite specific promoter is sufficient to control the stage specific expression of a reporter gene.

The microarray data of *Toxoplasma* merozoites provide a global gene expression dataset for this stage. Not only will this information be valuable in understanding the life cycle of *Toxoplasma*, it can be used to develop reagents and tools to further characterize the developmental biology of the sexual stages of coccidian parasites. For example, the transgenic parasites developed in this study, which express drug selectable markers only at the merozoite stage, will be used in forward selection strategies to screen for tissue culture conditions that are favorable to merozoite growth. As the merozoite is the first stage the parasite differentiates into within the gut of the definitive host, it is the first hurdle to better understand the sexual stages of the parasite. If we can determine the correct conditions that coax the parasite into the merozoite stage *in vitro*, those conditions may be sufficient to allow the parasite to complete sexual development.

Epithelial gut stages are a common life cycle feature of apicomplexan parasites, and most often the sexual stages develop in the gut, whether the host be vertebrate or invertebrate. This shared tropism is the result of the passive route of ingestion with which the parasite can gain access to and exit from the host. The triggers and specific processes that control parasite differentiation within gut environments are relatively unknown for apicomplexan parasites. In large part, this is because there are no tissue culture systems for gut stages. For example, it is necessary to use mosquitoes to perform crosses in *Plasmodium* [50] and to use chickens to grow *Eimeria tenella* [51]. It is not for lack of trying that efforts haven’t been successful in the past, but with the advent of -omic technologies these efforts can be re-explored. Using genomic based technologies the unique molecular characteristics of gut stages can be acquired and assessed for possible drivers of differentiation. This strategy has recently led to a major advance in understanding the tsetse fly specific developmental stages of *Trypanosome brucei*. Analysis of transcriptional data allowed researchers to show for the first time that the expression of just one RNA-binding protein in procyclics induced them *in vitro* into long and short form epimastigotes and eventually infectious metacyclics [52]. This approach has the potential to work in other systems, such as *Toxoplasma* that has many tools already developed for study, and may reveal unique and/or shared aspects of apicomplexan gut stage development.

## Conclusions

Merozoites are the first developmental stage in the coccidian cycle that takes place within the gut of the definitive host. The data presented here describe the global gene expression profile of the merozoite stage and the creation of transgenic parasite strains that show stage-specific expression of reporter genes in the cat intestine. These data and reagents will be useful in unlocking how the parasite senses and responds to the felid gut environment to initiate enteric development.

## Methods

### Ethics statement

Laboratory mice were used for maintaining chronic infections of the parasite *T. gondii*. Mice were housed according to instructions in the “Guide to Care and Use of Laboratory Animals” under supervision of a fully trained, veterinary staff in the Washington University Animal Care Facility. Protocols were approved by the Institutional Care Committee and are covered by animal welfare assurance number A-3381-01.

Members of the cat family are the only known host for the sexual stages of *T. gondii.* Protocols were conducted in the laboratory of Dr. J. P. Dubey at the USDA in Beltsville MD. Dr. Dubey’s laboratory is approved for these procedures by USDA, ARS, Beltsville Agricultural Research Center Animal Care Committee (BAACUC) and are covered by animal welfare assurance number A4400-01.

### Parasite lines and tissue culture

The type II TgNmBr1 strain was provided by J.P. Dubey [25]. The type II Me49-Fudr strain was used to make transgenic parasites [53]. For tissue culture, parasites were grown in human foreskin fibroblasts (HFFs) in Dulbecco’s Modified Eagle’s Medium (DMEM) (Gibco) supplemented with 10% fetal bovine serum (FBS) (HyClone) at 37°C with 5% CO_2_. For the plaque assays, parasites were seeded to confluent HFF monlayers at either 100 or 2000 per well and allowed to grow for 9 days (+/-pyrimethamine (Sigma), 10 μM), at which time the monolayers were washed with PBS, fixed with 100% ethanol and stained with Crystal Violet (.05%).

The TgNmBr1 parasite was grown in tissue culture as tachyzoites for mRNA isolation. Two day infected HFF monolayers were scrapped, needle passed, and parasites filtered through a 3 μm polycarbinate membrane (GE). Filtered parasites were spun at 1,410 rpm and parasite pellets were frozen at -80°C until processed for mRNA.

### Sexual stage parasite production and harvest

To produce intraepithelial intestinal sexual stage parasites, the TgNmBr1 strain was intraperitoneally (i.p.) injected into mice that were allowed to develop chronic infections characterized by tissue cysts containing bradyzoites. After 30 days, brains from the infected mice were harvested and fed to three separate cats (c48, c50, and c52). Production of oocysts was monitored, and once oocysts were detected (5–6 days post feeding), the small intestine was harvested and placed in 1X PBS with antibiotics and kept at 4°C. To harvest sexual stage parasites, intestines were flayed open, lightly scrapped to remove the mucus and first layer of intestinal cells, and the scrapped material was suspended in PBS. Samples were passed through progressively smaller needles (16G – 23G) to break host cells and debris, and centrifuged in an Eppendorf 5810R centrifuge at 400 rpm to remove large material. The supernatant was filtered through 3 μm membranes, centrifuged at 1,410 rpm to pellet parasites, and parasite pellets were frozen at -80°C until processed for RNA. A portion of each sample was resuspended in PBS and observed for purity under the microscope. The harvesting process resulted in removing the majority of host cells and debris, leaving crescent shaped felid intestinal derived parasites.

To test enteric stage expression of the MSF promoter, the transgenic Me49-Fudr-MSFp-DHFR-Ty parasite was i.p. injected into CD-1 mice to generate *in vivo* bradyzoite cysts. After 30 days, mice were sacrificed and cysts were isolated from brain samples and kept on ice until fed to a cat. The cat was monitored for oocyst shedding, and once oocysts were observed, the intestine was harvested, fixed in 10% formalin, and processed for IHC.

### RNA isolation and microarray hybridization

The Qiagen RNeasy kit (Qiagen) was used to harvest RNA as described previously [28]. Briefly, frozen parasite pellets were resuspended in RLT buffer with β-mercaptoethanol and processed on the RNeasy column. Samples were treated with DNase and resuspended in deionized water and frozen at -80°C. RNA quality was determined on an Agilent Bioanalyzer (Agilent Technologies). The RNA quality was good, showing lack of degradation and little host contamination. Two samples for the c52 RNA, one each for the c48 and c50 RNA, and two for tachyzoite TgNmBr1 RNA were labeled using the Ambion MessageAmp Premier RNA Amplification Kit (Life Technologies) using 500 ng total RNA. Using standard hybridization protocols, 5.5 μg of labeled cRNA was hybridized to the Affymetrix Toxoplasma GeneChip [26] and imaged with an Affymetrix GeneChip Scanner (Affymetrix). The microarray CEL files created for this study have been deposited at NCBI GEO submission GSE51780.

### Microarray data processing and analysis

To analyze the merozoite gene expression in context of the life cycle we obtained a previously published set of arrays from NCBI GEO GSE32427 [27], comprising the following sample types; day 0, 4, 10 oocyst (OD0, OD4, OD10), day 2 tachyzoite (TD2), and day 4, 8, 21 bradyzoite (BD4, BD8, BD21). Arrays from GSE32427 and those hybridized for this study (TNm, Mc48, Mc50, Mc52), GSE51780, were combined and analyzed as a set. Microarray data were loaded into R using the “affy” library and processed using Robust Multi-array Average (RMA) and quantile normalization [54]. RMA normalization converged the distributions and medians across all samples. RMA normalized data were used in subsequent analyses. The log_2_ RMA normalized intensity values can be acquired from NCBI GEO submission GSE51780. Annotations and Gene Ontology (GO) assignments were obtained from ToxoDB.org [55].

R normalized values were imported into GeneSpring (Agilent Technologies). Duplicate samples were grouped by type and average values were calculated. To identify those 8,131 Toxoplasma probesets, or genes, represented on the microarray with significant gene expression differences across sample types we used the following criteria: 1. genes with raw expression values of 30 or lower in all samples were flagged as not expressed and removed from analysis. This removed 1571 genes. 2. An analysis of variance (ANOVA) test was run on the remaining 6560 genes using a p-value cutoff of .05 and multiple testing correction: Benjamini and Hochberg False Discovery Rate. There were 43 genes that had insufficient data. This procedure identified 5969 genes significantly different in at least one sample across the experiment, of which 5% (298) may have been called by chance.

Heatmaps were generated using gene tree clustering with the standard correlation similarity measure.

PCA: Principle component analysis (PCA) was carried out on the 5,969 gene set by sample type, or condition, to determine the strongest expression themes in the data. This method calculates the standard correlation between each condition vector and each eigenvector, or principal component vector. PCA identified three principal components (PCA1: 43% variance, PCA2: 32.2% variance, PCA3: 10% variance), together these comprise 85% of the variance across sample types.

Spline curves: To accurately describe expression profiles across the life cycle for the 5,969 significantly differentially expressed genes we used the spline function in R. The TNm, BD4, BD21, Mc48, and Mc50 samples were not included in spline analysis as they are correlative to either the TD2, BD8, Mc52 samples. The excluded samples essentially represent replicate sample types for many genes that would hinder the correct determination of maximum and minimum spline values, confounding the ordering or grouping of genes in context of the life cycle. A spline curve was generated for each of the non-redundant 5969 genes using a “n” number of points 20*length (dataset) and the “natural” spline method. The global maximum and minimum, or extrema, spline values for each gene were determined, which correspond to points of maximum and minimum expression across the life cycle for a particular gene. The spline function was also used to determine the extrema of the first derivative, *f’(x)*, using “splinefun” and “deriv = 1”. The extrema of the first derivative are the inflection points along the spline curve that correspond to the maximum rate of synthesis or degradation for a particular gene. The differences between maximum, or minimum, expression and the respective rate values were calculated to determine the time to maximum or minimum expression.

FIRE: To identify putative *cis* elements in the 5’ regions of co-regulated genes across the life cycle we used the Finding Informative Regulatory Elements (FIRE) program [41]. Sequence 2,000-0 bp 5′ of each gene was obtained from ToxoDB.org [55] and combined with the continuous dataset of ordered genes according to the maximum or minimum of expression. A minimum cutoff z-score of 8.5 was used and motifs were sorted by phase of expression. No significant motifs were found for 100 permutations of randomly ordered genes. Also, no significant motifs were found for correctly ordered maximum of expression genes and only one significant motif was found for minimum of expression genes when compared to sequence 4,000-2,000 bp 5′ of each gene, and no significant motifs were found when correctly ordered genes were compared to sequence 6,000-4,000 bp 5′ of each gene.

KEGG: Normalized ratio expression values were used to map genes and expression into the Kyoto Encyclopedia of Genes and Genomes (KEGG) [56]. The *Toxoplasma gondii* genome is represented at KEGG, entry T01093, and life cycle regulated genes with KEGG assignments were mapped onto the PathwayMap using the KegArray program, pathways 2 fold downregulated (blue), non-regulated (yellow), and 2 fold upregulated (red). Images for the Metabolic Pathways map (tgo01100) were obtained for each sample and a GIF file was created using the convert program.

### Immunofluorescence, immunohistochemistry and microscopy

For IFA: Extracellular merozoite parasites were adhered to poly-L-lysine treated coverslips. Intracellular parasites were imaged on coverslips with infected HFF monolayers. All coverslips were fixed with 4% formaldehyde with .01% TritonX-100 in 1X PBS. Coverslips were blocked with 5% Fetal-bovine/Normal goat serum (FBS/NGS) in PBS. Coverslips were mounted in Prolong Gold containing DAPI (Life Technologies).

For IHC: Felid intestines were fixed in 10% formalin and stored in 70% EtOH. Samples were mounted in paraffin tissue blocks and thin sectioned.

For IFA and IHC: Antibodies were suspended in 1X PBS with 1% FBS. Primary antibodies used were all diluted 1:1,000 and include; mouse α-Me49, rabbit α-RH, mouse α-Ty, conjugated α-SAG1-594, and rabbit α-BAG5 (BAG5 is an alternate name for BAG1). Alexa Fluor 488 α-mouse or Alexa Fluor 595 α-rabbit (Life Technologies) were used for the secondary at 1:1000. Images were obtained on a Zeiss Axioskop 2 MOT Plus microscope using a AxioCam MRm camera (Carl Zeiss, Inc.).

### Plasmid construction and creation of transgenic parasites

To construct the merozoite promoter expression plasmids we used the pDEST-GRA1p as a template. The pDEST-GRA1p plasmid has a Destination Gateway cloning site flanked by an upstream TgGRA1 promoter (GRA1p) and a downstream TgDHFR 3′ UTR, and contains a bleomycin cassette for selection in *Toxoplasma*. The GRA1p (611 bp) was excised from pDEST-GRA1p using the Hind-III and Bgl-II restriction enzymes and replaced with either the MSF (998 bp upstream of the start codon) or the SRS22B (672 bp upstream) promoters that were amplified from type II Me49 lysate using the iProof polymerase (Bio-Rad Laboratories) with standard PCR amplification techniques, resulting in pDEST-MSFp and pDEST-SRS22Bp plasmids. Primers used to amplify the merozoite promoters: Megap Hind-III for (1) (5′-CTAGTAAGCTTTCTCCCCTGGGAAAAGACAGG-3′), Megap Bgl-II rev (5′-CTAGTAGATCTGCCGTTTTGGTGCGTCCAAG-3′), SRS22Bp Hind-III for (1) (5′-CTAGTAAGCTTCTGTGCGTCCTCCACCTTC-3′), SRS22Bp Bgl-II rev (5′-CTAGTAGATCTCTTGAATTAACTGAGACCAGGGCCAC-3′). To create a Gateway cloning fragment for the pDEST plasmids, the pyrimethamine resistant DHFR gene was cloned into the pDONR221 plasmid with a C-terminal Ty tag using the standard BP reaction protocol, resulting in pDONR-DHFR-Ty. Primers used to clone pDONR-DHFR-Ty: DHFR-Ty CDS attB1 for (5′-GGGGACAAGTTTGTACAAAAAAGCAGGCTTCATGCAGAAACCGGTGTGTCTGG-3′), DHFR-Ty CDS attB2 rev (5′- GGGGACCACTTTGTACAAGAAAGCTGGGTCATCGAGCGGGTCCTGGTTCGTGTGGACCTCGACAGCCATCTCCATCTGGA -3′). The pDONR-DHFR-Ty plasmid was then cloned into the three pDEST plasmids using the LR reaction to create, pDEST-GRA1p-DHFR-Ty, pDEST-MSFp-DHFR-Ty, and pDEST-SRS22Bp-DHFR-Ty. The three plasmids were individually electroporated into Me49-Fudr, parasites were selected with phloemycin using standard protocols [57], cloned, and screened for plasmid integration using specific primers (See Figure 6A). Primers used to screen clones (for (1), see above): GRA1p for (G1) (5′- AAACCCTCGAAGGCTGCTAGTACT 3′), GRA1p rev (G2) (5′- TCTTGCTTGATTTCTTCAAAGAACAACAGCAAG-3′), DHFR-Ty in 5′ of CDS rev (2) (5′- CCCGTCTTTGCAAATTTCCTGG-3′), DHFR 3′ UTR rev (3) (5′- CCGCGGTGTCACTGTAGCC -3′). This resulted in three transgenic strains; Me49-GRA1p-DHFR-Ty (GRA1 strain), Me49-MSFp-DHFR-Ty (MSF strain) and Me49-SRS22Bp-DHFR-Ty (SRS22B strain).

### Availability of supporting data

Microarray CEL files and log_2_ normalized intensity values are available at NCBI GEO, http://www.ncbi.nlm.nih.gov/geo/, under Accession number GSE51780.

## Electronic supplementary material

Additional file 1: **KEGG maps.** Description: KEGG maps for life cycle regulated genes were created for each of the microarray samples and combined together into a GIF file. Expression of genes that mapped to a particular metabolic pathway are indicated by colored lines; 2 fold downregulated (blue), non-regulated (yellow), and 2 fold upregulated (red), gray lines are pathways without a mapped gene. (GIF 3 MB)

Additional file 2: **Raw Expression Data.** Description: Excel file with raw expression values for all genes across all samples used in this study. Genes are grouped by expression type, color coding denoted at the top of the file. (XLSX 1 MB)

## Abbreviations

ANOVA: Analysis of Variance
PCA: Principal component analysis
IFA: Immunofluorescence assay
IHC: Immunohistochemistry
f’(x): First derivative
FIRE: Finding Informative Regulatory Elements
MSF: Megakaryocyte Stimulating Factor.

## References

[1] Montoya JG, Liesenfeld O (2004). Toxoplasmosis. Lancet.

[2] Jones JL, Roberts JM (2012). Toxoplasmosis hospitalizations in the United States, 2008, and trends, 1993–2008. Clin Infect Dis.

[3] Jones JL, Dargelas V, Roberts J, Press C, Remington JS, Montoya JG (2009). Risk factors for Toxoplasma gondii infection in the United States. Clin Infect Dis.

[4] Jones JL, Holland GN (2010). Annual burden of ocular toxoplasmosis in the US. Am J Trop Med Hyg.

[5] Scallan E, Hoekstra RM, Angulo FJ, Tauxe RV, Widdowson MA, Roy SL, Jones JL, Griffin PM (2011). Foodborne illness acquired in the United States–major pathogens. Emerg Infect Dis.

[6] Jones JL, Kruszon-Moran D, Sanders-Lewis K, Wilson M (2007). Toxoplasma gondii infection in the United States, 1999 2004, decline from the prior decade. Am J Trop Med Hyg.

[7] Dubey JP (2009). History of the discovery of the life cycle of Toxoplasma gondii. Int J Parasitol.

[8] Hill DE, Chirukandoth S, Dubey JP (2005). Biology and epidemiology of Toxoplasma gondii in man and animals. Anim Health Res Rev.

[9] Su C, Evans D, Cole RH, Kissinger JC, Ajioka JW, Sibley LD (2003). Recent expansion of Toxoplasma through enhanced oral transmission. Science.

[10] Dubey JP, Kreier JP (1977). *Toxoplasma, Hammondia, Besniotia, Sarcocystis*, and Other Tissue Cyst-forming Coccidia of Man and Animals. Parasitic Protozoa.

[11] Dubey JP (2008). The history of Toxoplasma gondii–the first 100 years. J Eukaryot Microbiol.

[12] Hogan MJ, Yoneda C, Feeney L, Zweigart P, Lewis A (1960). Morphology and culture of Toxoplasma. Trans Am Ophthalmol Soc.

[13] Matsubayashi H, Akao S (1963). Morphological studies on the development of the Toxoplasma cyst. Am J Trop Med Hyg.

[14] Weiss LM, Laplace D, Takvorian PM, Tanowitz HB, Cali A, Wittner M (1995). A cell culture system for study of the development of Toxoplasma gondii bradyzoites. J Eukaryot Microbiol.

[15] Soete M, Camus D, Dubremetz JF (1994). Experimental induction of bradyzoite-specific antigen expression and cyst formation by the RH strain of Toxoplasma gondii in vitro. Exp Parasitol.

[16] Sibley LD (2011). Invasion and intracellular survival by protozoan parasites. Immunol Rev.

[17] Boyle JP, Radke JR (2009). A history of studies that examine the interactions of Toxoplasma with its host cell: Emphasis on in vitro models. Int J Parasitol.

[18] Hunter CA, Sibley LD (2012). Modulation of innate immunity by Toxoplasma gondii virulence effectors. Nat Rev Microbiol.

[19] Dubey JP, Frenkel JK (1972). Cyst-induced toxoplasmosis in cats. J Protozool.

[20] Ferguson DJ (2004). Use of molecular and ultrastructural markers to evaluate stage conversion of Toxoplasma gondii in both the intermediate and definitive host. Int J Parasitol.

[21] Ferguson DJ, Hutchison WM, Dunachie JF, Siim JC (1974). Ultrastructural study of early stages of asexual multiplication and microgametogony of Toxoplasma gondii in the small intestine of the cat. Acta Pathol Microbiol Scand B: Microbiol Immunol.

[22] Speer CA, Dubey JP (2005). Ultrastructural differentiation of Toxoplasma gondii schizonts (types B to E) and gamonts in the intestines of cats fed bradyzoites. Int J Parasitol.

[23] Omata Y, Taka A, Terada K, Koyama T, Kanda M, Saito A, Dubey JP (1997). Isolation of coccidian enteroepithelial stages of Toxoplasma gondii from the intestinal mucosa of cats by Percoll density-gradient centrifugation. Parasitol Res.

[24] Taka A, Omata Y, Ohsawa T, Koyama T, Kanda M, Saito A, Toyoda Y (1999). Antibody reactivity in mice and cats to feline enteroepithelial stages of Toxoplasma gondii. Vet Parasitol.

[25] Dubey JP, Passos LM, Rajendran C, Ferreira LR, Gennari SM, Su C (2011). Isolation of viable Toxoplasma gondii from feral guinea fowl (Numida meleagris) and domestic rabbits (Oryctolagus cuniculus) from Brazil. J Parasitol.

[26] Bahl A, Davis PH, Behnke M, Dzierszinski F, Jagalur M, Chen F, Shanmugam D, White MW, Kulp D, Roos DS (2010). A novel multifunctional oligonucleotide microarray for *Toxoplasma gondii*. BMC Genomics.

[27] Fritz HM, Buchholz KR, Chen X, Durbin-Johnson B, Rocke DM, Conrad PA, Boothroyd JC (2012). Transcriptomic analysis of toxoplasma development reveals many novel functions and structures specific to sporozoites and oocysts. PLoS One.

[28] Behnke MS, Radke JB, Smith AT, Sullivan WJ, White MW (2008). The transcription of bradyzoite genes in Toxoplasma gondii is controlled by autonomous promoter elements. Mol Microbiol.

[29] Cleary MD, Singh U, Blader IJ, Brewer JL, Boothroyd JC (2002). Toxoplasma gondii asexual development: identification of developmentally regulated genes and distinct patterns of gene expression. Eukaryot Cell.

[30] Radke JR, Behnke MS, Mackey AJ, Radke JB, Roos DS, White MW (2005). The transcriptome of Toxoplasma gondii. BMC Biol.

[31] Samuelson J, Bushkin GG, Chatterjee A, Robbins PW (2013). Strategies to discover the structural components of cyst and oocyst walls. Eukaryot Cell.

[32] Braun L, Brenier-Pinchart MP, Yogavel M, Curt-Varesano A, Curt-Bertini RL, Hussain T, Kieffer-Jaquinod S, Coute Y, Pelloux H, Tardieux I, Sharma A, Belrhali H, Bougdour A, Hakimi MA (2013). A Toxoplasma dense granule protein, GRA24, modulates the early immune response to infection by promoting a direct and sustained host p38 MAPK activation. J Exp Med.

[33] Carruthers VB, Tomley FM (2008). Microneme proteins in apicomplexans. Subcell Biochem.

[34] Wasmuth JD, Pszenny V, Haile S, Jansen EM, Gast AT, Sher A, Boyle JP, Boulanger MJ, Parkinson J, Grigg ME (2012). Integrated bioinformatic and targeted deletion analyses of the SRS gene superfamily identify SRS29C as a negative regulator of Toxoplasma virulence. MBio.

[35] Behnke MS, Wootton JC, Lehmann MM, Radke JB, Lucas O, Nawas J, Sibley LD, White MW (2010). Coordinated progression through two subtranscriptomes underlies the tachyzoite cycle of Toxoplasma gondii. PLoS One.

[36] Bozdech Z, Llinas M, Pulliam BL, Wong ED, Zhu J, DeRisi JL (2003). The transcriptome of the intraerythrocytic developmental cycle of Plasmodium falciparum. PLoS Biol.

[37] Radke JR, Gubbels MJ, Jerome ME, Radke JB, Striepen B, White MW (2004). Identification of a sporozoite-specific member of the Toxoplasma SAG superfamily via genetic complementation. Mol Microbiol.

[38] Bohne W, Wirsing A, Gross U (1997). Bradyzoite-specific gene expression in Toxoplasma gondii requires minimal genomic elements. Mol Biochem Parasitol.

[39] Nakaar V, Bermudes D, Peck KR, Joiner KA (1998). Upstream elements required for expression of nucleoside triphosphate hydrolase genes of Toxoplasma gondii. Mol Biochem Parasitol.

[40] Soldati D, Boothroyd JC (1993). Transient transfection and expression in the obligate intracellular parasite Toxoplasma gondii. Science.

[41] Elemento O, Slonim N, Tavazoie S (2007). A universal framework for regulatory element discovery across all genomes and data types. Mol Cell.

[42] Van Poppel NF, Welagen J, Vermeulen AN, Schaap D (2006). The complete set of Toxoplasma gondii ribosomal protein genes contains two conserved promoter elements. Parasitology.

[43] Walker R, Gissot M, Huot L, Alayi TD, Hot D, Marot G, Schaeffer-Reiss C, Van Dorsselaer A, Kim K, Tomavo S (2013). Toxoplasma transcription factor TgAP2XI-5 regulates the expression of genes involved in parasite virulence and host invasion. J Biol Chem.

[44] Goodswen SJ, Kennedy PJ, Ellis JT (2013). A review of the infection, genetics, and evolution of Neospora caninum: from the past to the present. Infect Genet Evol.

[45] David Sibley L (2003). Recent origins among ancient parasites. Vet Parasitol.

[46] Piekarski G, Pelster B, Witte HM (1971). Endopolygeny in Toxoplasma gondii. Z Parasitenkd.

[47] Cairns RA, Harris IS, Mak TW (2011). Regulation of cancer cell metabolism. Nat Rev Cancer.

[48] Colgan SP, Taylor CT (2010). Hypoxia: an alarm signal during intestinal inflammation. Nat Rev Gastroenterol Hepatol.

[49] Radke JB, Lucas O, De Silva EK, Ma Y, Sullivan WJ, Weiss LM, Llinas M, White MW (2013). ApiAP2 transcription factor restricts development of the Toxoplasma tissue cyst. Proc Natl Acad Sci U S A.

[50] Baker DA (2010). Malaria gametocytogenesis. Mol Biochem Parasitol.

[51] Muller J, Hemphill A (2013). In vitro culture systems for the study of apicomplexan parasites in farm animals. Int J Parasitol.

[52] Kolev NG, Ramey-Butler K, Cross GA, Ullu E, Tschudi C (2012). Developmental progression to infectivity in Trypanosoma brucei triggered by an RNA-binding protein. Science.

[53] Behnke MS, Khan A, Wootton JC, Dubey JP, Tang K, Sibley LD (2011). Virulence differences in Toxoplasma mediated by amplification of a family of polymorphic pseudokinases. Proc Natl Acad Sci U S A.

[54] Gautier L, Cope L, Bolstad BM, Irizarry RA (2004). affy–analysis of Affymetrix GeneChip data at the probe level. Bioinformatics.

[55] Gajria B, Bahl A, Brestelli J, Dommer J, Fischer S, Gao X, Heiges M, Iodice J, Kissinger JC, Mackey AJ, Pinney DF, Roos DS, Stoeckert CJ, Wang H, Brunk BP (2008). ToxoDB: an integrated Toxoplasma gondii database resource. Nucleic Acids Res.

[56] Kanehisa M, Goto S, Sato Y, Furumichi M, Tanabe M (2012). KEGG for integration and interpretation of large-scale molecular data sets. Nucleic Acids Res.

[57] Messina M, Niesman I, Mercier C, Sibley LD (1995). Stable DNA transformation of Toxoplasma gondii using phleomycin selection. Gene.

[58] Sibley LD, Khan A, Ajioka JW, Rosenthal BM (2009). Genetic diversity of Toxoplasma gondii in animals and humans. Philos Trans R Soc Lond B Biol Sci.

