# Supplementary figures and images for: Toxoplasma gondii merozoite gene expression analysis with comparison to the life cycle discloses a unique expression state during enteric development
Source: BMC Genomics. 2014 May 8;15(1):350. doi: 10.1186/1471-2164-15-350 (PMC4035076; doi:10.1186/1471-2164-15-350)

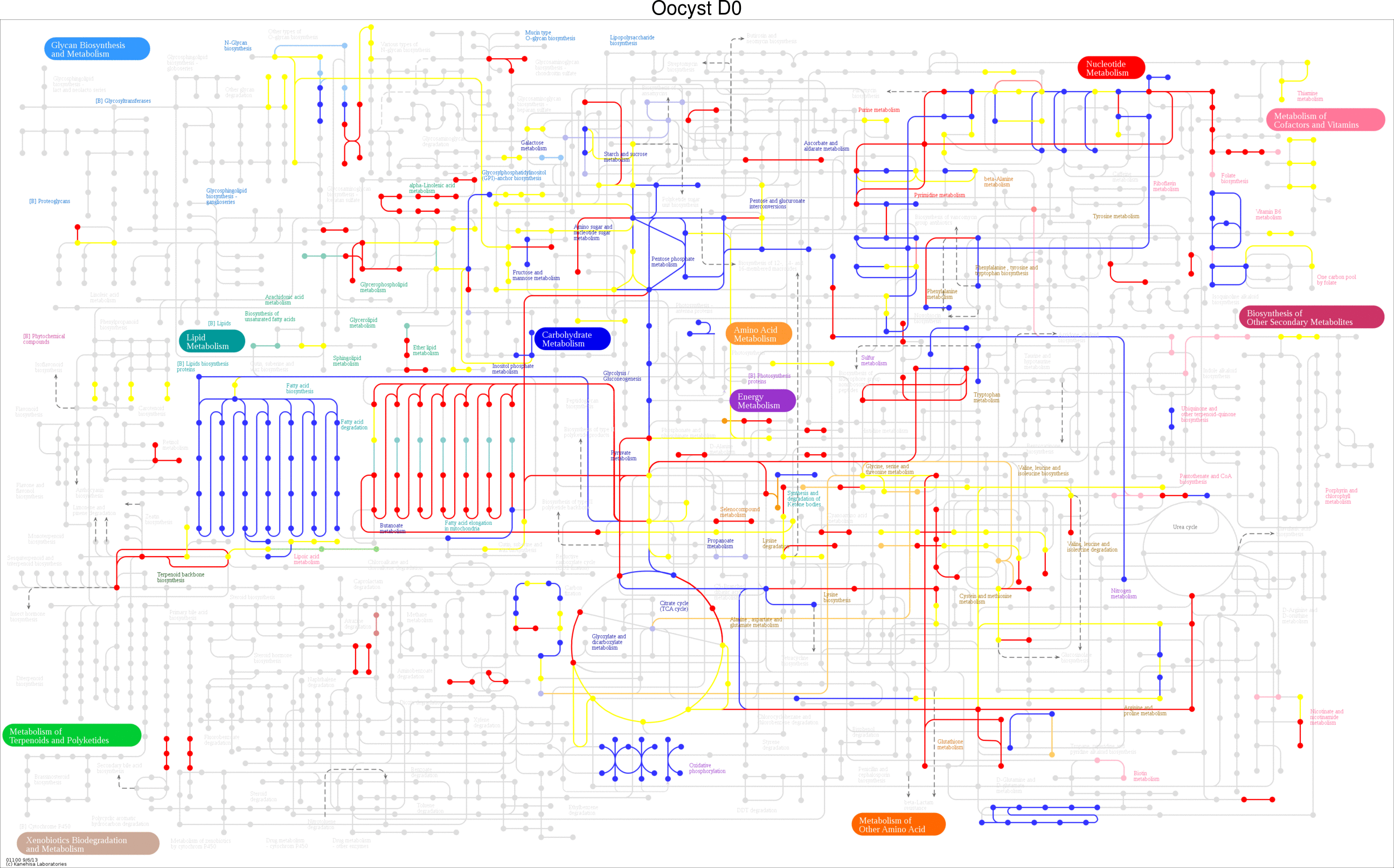

Supplement: Supplementary file 1 — Additional file 1: KEGG maps. Description: KEGG maps for life cycle regulated genes were created for each of the microarray samples and combined together into a GIF file. Expression of genes that mapped to a particular metabolic pathway are indicated by colored lines; 2 fold downregulated (blue), non-regulated (yellow), and 2 fold upregulated (red), gray lines are pathways without a mapped gene. (GIF 3 MB) [file 12864_2013_6044_MOESM1_ESM.gif]
